# Clinicians’ knowledge and practices regarding family planning and intrauterine devices in China, Kazakhstan, Laos and Mexico

**DOI:** 10.1186/s12978-016-0185-1

**Published:** 2016-06-10

**Authors:** Steven J. Hoffman, G. Emmanuel Guindon, John N. Lavis, Harkanwal Randhawa, Francisco Becerra-Posada, Boungnong Boupha, Guang Shi, Botagoz S. Turdaliyeva, for the Research to Policy & Practice Study Team, John N. Lavis, G. Emmanuel Guindon, David Cameron, Steven J. Hoffman, Guang Shi, Tinglin Qiu, Eric J. A. Osei, Kudjoe Dovlo, C. A. K. Yesudian, P. Ramachandran, Hossein Malek-Afzali, M. Dejman, K. Falahat, M. Baradaran, E. Habibi, H. Kohanzad, M. Nasehi, S. Salek, A. A. Akanov, B. S. Turdaliyeva, N. K. Hamzina, K. A. Tulebaev, T. I. Clazhneva, G. Battakova, Boungnong Boupha, Sengchanh Kounnavong, Latsamy Siengsounthone, Francisco Becerra-Posada, Leticia Alfaro Ramos, Israel Mejia, Tasleem Akhtar, M. Mubashir A. Khan, Mintou Fall Sidibe, Awa Sidibe, Djiby Ndiaye, Godwin D. Ndossi, Julius Massaga, Ritu Sadana, Tikki Pang

**Affiliations:** Global Strategy Lab, Centre for Health Law, Policy & Ethics, Faculty of Law, University of Ottawa, Fauteux Hall, 57 Louis Pasteur Street, Ottawa, ON K1N 6N5 Canada; Department of Clinical Epidemiology & Biostatistics, McMaster University, Hamilton, ON Canada; McMaster Health Forum, McMaster University, Hamilton, ON Canada; Department of Global Health & Population, Harvard T.H. Chan School of Public Health, Harvard University, Boston, MA USA; Centre for Health Economics & Policy Analysis, McMaster University, Hamilton, ON Canada; Pan American Health Organization, Washington, DC USA; Foreign Affairs Committee and Women’s Caucus, Laos National Assembly, Vientiane, Lao PDR; Democratic Party of Peasants & Workers in China, Beijing, China; Department of Health Policy & Management, Kazakh National Medical University, Almaty, Kazakhstan; Evidence-Based Health Centre, Almaty, Kazakhstan

**Keywords:** Family planning, Intrauterine device, Global health, Knowledge translation, Health professionals, Medical education, Systematic reviews, Health systems, Health human resources

## Abstract

**Background:**

It is widely agreed that the practices of clinicians should be based on the best available research evidence, but too often this evidence is not reliably disseminated to people who can make use of it. This “know-do” gap leads to ineffective resource use and suboptimal provision of services, which is especially problematic in low- and middle-income countries (LMICs) which face greater resource limitations. Family planning, including intrauterine device (IUD) use, represents an important area to evaluate clinicians’ knowledge and practices in order to make improvements.

**Methods:**

A questionnaire was developed, tested and administered to 438 individuals in China (*n* = 115), Kazakhstan (*n* = 110), Laos (*n* = 105), and Mexico (*n* = 108). The participants responded to ten questions assessing knowledge and practices relating to contraception and IUDs, and a series of questions used to determine their individual characteristics and working context. Ordinal logistic regressions were conducted with knowledge and practices as dependent variables.

**Results:**

Overall, a 96 % response rate was achieved (*n* = 438/458). Only 2.8 % of respondents were able to correctly answer all five knowledge-testing questions, and only 0.9 % self-reported “often” undertaking all four recommended clinical practices and “never” performing the one practice that was contrary to recommendation. Statistically significant factors associated with knowledge scores included: 1) having a masters or doctorate degree; and 2) often reading scientific journals from high-income countries. Significant factors associated with recommended practices included: 1) training in critically appraising systematic reviews; 2) training in the care of patients with IUDs; 3) believing that research performed in their own country is above average or excellent in quality; 4) being based in a facility operated by an NGO; and 5) having the view that higher quality available research is important to improving their work.

**Conclusions:**

This analysis supports previous work emphasizing the need for improved knowledge and practices among clinicians concerning the use of IUDs for family planning. It also identifies areas in which targeted interventions may prove effective. Assessing opportunities for increasing education and training programs for clinicians in research and IUD provision could prove to be particularly effective.

## Background

The healthcare community, including its numerous researchers, clinicians, and policymakers, have become increasingly aware of the worrisome gap between what we know from research evidence and what is brought into action [1, 2]. A growing body of work supports the notion that clinicians are not regularly receiving research results, leading to a troubling information gap which can result in inadequate and inequitable care, suboptimal resource allocation, and an overall decreased quality of healthcare services. Further, this problem is exacerbated in low- and middle-income countries (LMICs) where resources are scarcer. This problematic trend is especially apparent in global health issues for which treatments exist but lack universally consistent action [3].

Maternal and reproductive health has been highlighted in both the Millennium Development Goals (MDGs) and Sustainable Development Goals (SDGs) because of its significant impact on quality of life in LMICs. A principal objective proposed by the World Health Organization (WHO) is to lower unmet contraception needs globally, not only in terms of improving access, but also with regard to increasing options available to women. Inequitable access and ineffective use of contraception persist as barriers to improving women’s health globally. Progress in this field would serve to reduce maternal, newborn and child mortality, while also empowering vulnerable populations through improvements in education and gender equality [4, 5]. Intrauterine devices (IUDs), which are long-acting reversible contraception products, have been proven to be safe over extended periods of use and shown to be nearly as effective as male or female sterilization [6]. With the demonstrated efficacy and cost-effectiveness of IUDs for family planning, there is a clear need to address any knowledge gaps and their underuse globally [7].

Additionally, the gap in accessible and affordable contraceptive options supports the need for further investigation in the realm of family planning and reproductive health services. The current state of contraceptive options poses a significant threat to global development [8]. Despite most maternal deaths being preventable, nearly 300,000 women die each year from causes related to pregnancy and childbirth [9]. The overwhelming number of unintended pregnancies worldwide contributes to this loss. For example, women in lower socioeconomic settings who do not have access to safe delivery services face high maternal mortality risks [10]. This palpable inequity requires an immediate response. Facilitating improvements in this area will require a deeper understanding of the knowledge and practices of family planning and reproductive clinicians to develop better-targeted strategies.

This exploratory study aims to investigate the gap between research evidence on IUDs and the knowledge and practices of clinicians in LMICs about this important family planning intervention. As clinicians have a significant influence on their patients’ health, wellbeing, and behaviour [11–15], appropriate clinical knowledge and clinician-patient interactions are necessary for achieving targeted health outcomes. Several studies have assessed the impact of clinician education on IUD use and other existing factors affecting IUD provision among clinicians [16–18]. Studies assessing clinicians’ knowledge and practices have illustrated that they may have limited or erroneous understandings of optimal IUD clinical use [19–27]. As such, this new study seeks to explore determinants of clinicians’ knowledge and practices surrounding IUD provision across sectors and in multiple countries. This research aims to highlight areas in family planning and reproductive healthcare that require attention and can be targeted through focused regional and global interventions.

## Methods

This study was one component of a larger WHO-sponsored research project that investigated the relationship between research, practice and policy. Other studies within this project have explored how clinicians use research evidence [28, 29], investigated the extent to which researchers support its use [30, 31], and evaluated clinicians’ knowledge and practices on malaria prevention [32] and tuberculosis treatment [33]. This study represents the first presentation of the collected data on clinicians’ knowledge and practices related to family planning and IUD provision.

### Questionnaire design

The questionnaire administered in this study was developed based on nine existing questionnaires [34–42]. Development and testing of the questionnaire showed it to have high internal consistency and good face and content validity. Details on its development, pilot-testing, assessment for validity and reliability, and translation have been documented elsewhere [28]. The survey tool incorporated questions addressing participants’ individual characteristics, working context, training, networking activities, and access to, trust in and use of research evidence. Additionally, five true/false questions were used to test respondents’ knowledge of IUDs for family planning and five other questions assessed relevant clinical practices [43]. The questionnaire was translated into Mandarin, Russian, Lao and Spanish by WHO’s translation service and local country teams [30].

### Data collection

Country teams administered the questionnaire in China, Kazakhstan, Laos and Mexico between October 2004 and December 2005. The teams were each led by one co-author (GS in China, BST in Kazakhstan, BB in Laos, and FBP in Mexico) and supported by the Research to Policy & Practice Study Team (all members listed in Acknowledgements section). The four selected LMICs differ in population size, per capita income, health expenditures, life expectancy, contraceptive prevalence, and Internet access (Table 1) [44–49]. Each team aimed to gather complete responses from a representative sample of at least 100 clinicians in each country (the vast majority of participants were physicians). The questionnaire was administered using a drop-off and pick-up approach (except in Mexico, where a mix of drop-off and pick-up and in-person administration was utilized). Various approaches to increase response rate were employed, including personalized letters and providing a free set of WHO publications as an incentive. More detailed information about the data collection process is described elsewhere [28, 29].

**Table 1:** Country profiles in 2005

	China	Kazakhstan	Laos	Mexico	Source
Population (in millions)	1323	15	6	107	[44]
GDP per capita (in PPP int'l $)	6771	8387	2147	10,626	[45]
Per capita total expenditure on health (in PPP int’l $)	277	264	74	655	[46]
Per capita government expenditure on health (in PPP int’l $)	105	158	15	304	[46]
Life expectancy at birth for males/females (in years)	71/74	59/71	61/63	72/77	[47]
Children under-five mortality rate (per 1, 000 live births)	27	73	79	27	[47]
Contraceptive prevalence among women using modern contraception methods among those of reproductive age (15–49) who are married or in a union (%)	85 (2006)	51 (2010/11)	38 (2005)	71 (2006)	[48]
Maternal Mortality Rate (maternal deaths per 100, 000 live births)	50	50	410	50	[49]
Internet users per population (%, 2004)	7	3	0	13	[47]

Data are for 2005 unless otherwise indicated

Systematic reviews are credited as the ideal method of synthesizing global research evidence and offering summary information to aid decision-makers. Furthermore, they are extensively available and internationally authoritative on clinical interventions like IUDs [50–52]. As a result, systematic reviews have been extensively used in this analysis, with the Cochrane Library representing the most comprehensive identified source. Efforts to distinguish between summaries and full reports, as well as between scientific journals from a high-income country and a participant’s own country, were guided by past studies [29, 36, 53].

In China, the team used a stratified random sampling process (with a sampling frame constructed from an existing list of family planning centres) to sample 120 clinicians who provided care to women seeking contraception. Stratification was by geographic location (Sichuan province in the southwest, and Liaoning province in the northeast) and by type of facility (Maternal and Child Health clinics, Departments of Obstetrics and Gynecology in hospitals, and Family Planning service institutions).

In Kazakhstan, a sampling frame was constructed from an existing database from the Department of Health of Almaty. The total population of gynaecologists (*n* = 110) that were working at the primary care level in family planning and contraceptive care in Almaty was sampled.

In Laos, a sampling frame was constructed from existing lists of clinicians retrieved from the Ministry of Health’s Department of Human Resources for Health and four provincial health departments, and was stratified by facility type (central, provincial and district hospital). 106 clinicians providing contraceptive care in the capital city of Vientiane and the provinces of Vientiane, Borikhamsay and Savannakheth were sampled.

In Mexico, the team used a sampling frame constructed from five sources (physicians working for the Mexican Foundation for Family Planning, physicians working for the Instituto de Seguridad y Servicios Sociales de los Trabajadores del Estado, physicians working for the Secretaria de Salud [Ministry of Health] in five facilities of Mexico City, physicians involved in family planning attending a training course at the Desarrollo Integral de la Familia and at the Instituto Mexicano del Seguro Social, and private physicians involved in family planning attending a training course). The sampling process was used to sample 122 clinicians in Mexico City and the states of México, Nuevo León and Jalisco.

### Data analysis

Using relevant items from the data collected, basic descriptive statistics were calculated and simple ordinal logistic regressions were conducted. The regression models were used to analyze the factors associated with clinicians’ knowledge and practices on IUD use and family planning. Composite knowledge and practice scores were constructed for each respondent and used as the dependent variables in the regression analyses. Knowledge scores were based on the proportion of the five true/false knowledge-testing questions that were correctly answered. Each question was assigned equal weight and no penalty was given for an incorrect answer. Similarly, practice scores were based on the frequency in which a clinician reported performing the five practices on a five-point scale (i.e., 1 “never”, 2 “rarely”, 3 “sometimes”, 4 “often”, and 5 “very often”). The scale was inverted for one described practice that is contrary to recommended practice. Each question held equal weight to ensure practice scores were integers that ranged from five to 25.

Knowledge and practice scores were converted to tertiles for inclusion as ordinal variables. For both knowledge and practice models, independent variables included clinicians’: 1) utilization of specific sources of research evidence; 2) views and behaviours related to the improvement of clinical practice; and 3) individual and practice characteristics. In order to substitute for missing values, multiple imputation using multivariate normal regressions was employed (with 100 imputations). Observations with missing dependent variables were omitted from the analysis. Stata/MP 11.2 for Mac was used to perform all statistical analyses [54].

## Results

Four hundred thirty eight of the 458 clinicians approached for the study provided complete responses, resulting in a 96 % overall response rate. Country-specific rates were 96 % in China (*n* = 115/120), 100 % in Kazakhstan (*n* = 110/110), 99 % in Laos (*n* = 105/106) and 89 % in Mexico (*n* = 108/122).

The majority of participating clinicians were female (80.6 %) and trained and practicing as physicians (89.6 %), especially general practitioners (63.6 %). Additionally, most participants worked in urban areas (68 %), government-operated facilities (95.4 %), and hospitals (50 %) or community health centres (44.2 %). Only a few respondents could read and write English (25.5 %) and had easy access to either a personal computer with a CD-ROM drive (23.4 %) or the Internet (16.1 %). Only a small fraction of the participants had earned masters or doctorate degrees (4.3 %). Almost one-third worked with researchers or research groups (29.6 %).

Participants were, on average, 41.8 years old and spent the largest portion of their time on clinical practice (75.7 %) versus research (5.1 %), teaching (7.1 %) and administrative duties (9.1 %). Very few respondents were trained on acquiring, assessing or adapting research evidence after their last degree, although a substantial number received training specifically related to contraceptive care (62.9 %). Additionally, only a few respondents self-reported using or reading the electronic Cochrane Library over the past 12 months (4.5 %). Nonetheless, more participants self-reported reading electronic or paper versions of clinical practice guidelines, protocols or decision-support tools (68.8 %), scientific journals from either their own country (79.7 %) or high-income countries (25.9 %), and summaries of articles, reports and reviews from public or non-profit organizations (46.6 %). More than half of the respondents reported that research performed in their own country was of above average or excellent quality (57.3 %), and the vast majority believed that a higher quality of available research is important or very important to improve their work (92.1 %) (Table 2).

**Table 2:** Descriptive statistics on participating clinicians’ individual characteristics, working context, and views about and use of research evidence

Factor	All (*n* = 438)	China (*n* = 115)	Kazakhstan (*n* = 110)	Laos (*n* = 105)	Mexico (*n* = 108)
Individual characteristics					
Age, yr, mean	41.8	39.8	38.8	41.2	47.8
Sex, female	80.6	93.9	99.1	84.8	42.3
Type of health professional					
General practitioner	63.6	1.7	97.3	78.1	81.7
Specialist physician	26.0	83.5	0.0	1.0	15.4
Nurse	5.3	6.1	0.9	14.3	0.0
Health worker	1.6	2.6	1.8	1.0	1.0
Other	3.5	6.1	0.0	5.7	1.9
Allocation of time, % of time^b^					
Clinical practice	75.7	80.9	83.4	68.0	68.9
Research	5.1	6.0	5.1	5.2	4.0
Teaching	7.1	6.5	1.3	11.7	9.8
Administration	9.1	6.3	6.8	12.2	11.7
Masters or doctorate degree	4.3	2.6	0.0	4.4	10.6
Training since completed last degree					
Acquiring systematic reviews through the Cochrane Library	4.5	2.6	8.0	2.9	7.0
Critically appraising systematic reviews	10.7	8.8	7.7	2.9	23.3
Care of women seeking contraception	62.9	64.9	76.9	55.8	58.5
Easy access to personal computer with CD ROM (v. less easy, not easy, no access or not sure)	23.4	35.1	13.4	8.6	34.8
Easy access to Internet (v. less easy, not easy, no access or not sure)	16.1	20.2	12.2	1.9	31.1
Able to read and write English well or very well (v. little or no ability)	25.5	34.8	10.9	20.4	35.6
Practice^a^					
Operating authority of facility or practice					
Government	95.4	99.1	96.3	100	85.6
Nongovernmental organization	4.6	0.0	4.6	0.0	14.4
For-profit organization	1.4	0.9	0.9	0.0	3.8
Type of facility or practice					
Solo or individual practice	11.3	0.0	4.5	13.3	29.4
Group practice	18.8	1.7	0.9	50.5	24.5
Hospital	50.0	85.2	7.3	96.2	8.8
Community health centre	44.2	0.0	92.7	22.9	63.7
Location of facility or practice					
Urban	68.0	45.2	87.9	54.3	86.5
Rural	2.1	2.6	0.0	2.9	2.9
Mixed	30.2	52.2	12.1	42.9	11.5
Facility had intrauterine devices (IUD) available	78.0	100	24.8	91.4	96.1
Views and activities related to improving clinical practice					
Research performed in their own country is of above average or excellent quality	57.3	84.3	55.8	19.4	67.0
Trust somewhat or completely a systematic review of randomized controlled double-blind trials	58.6	78.1	48.8	35.6	68.4
Working with researchers or research groups to improve clinical practice or the quality of working life	29.6	30.7	36.6	35.9	15.7
Higher quality of available research is important or very important to improve their work	92.1	86.7	97.0	92.2	93.1
Used or read particular sources of evidence					
Clinical practice guidelines, protocols or decision-support tools	68.8	81.4	91.1	42.6	62.5
Cochrane Library	4.5	5.3	2.7	2.9	6.9
Scientific journals from high-income countries	25.9	18.6	32.2	12.9	46.5
Scientific journals from own country	79.7	92.2	98.0	57.0	69.5
Summaries of articles, reports, and reviews from public and not-for-profit health organizations	46.6	34.5	60.8	37.0	60.2

^a^May not add to 100 % because health professional may practise in more than one setting

^b^May not add to 100 % because the allocation of time reported by a small number of respondents did not add to 100 %

Note that because of variations among sampling frames and a limited sample size, these results cannot, and should not, be compared across countries

Very few participating clinicians correctly responded to all five knowledge-testing questions about contraception and IUD use (2.8 %), with country-specific rates of 0.9 % in China, 6.4 % in Kazakhstan, 3.8 % in Laos and 5.8 % in Mexico. The overall correct response rate for individual questions ranged from 29.5 % on the sufficiency of one follow-up visit after the first menses or 3–6 weeks following copper-bearing IUD insertion (Question D), to 74.5 % on the question of whether spotting or light bleeding between menstrual periods during the first 3–6 months of copper-bearing IUD use is common or harmful (Question B). The range of overall correct responses to individual questions varied the greatest within China (9–98 %) and the least within Kazakhstan (57–87 %). Only 34.4 % of the respondents knew that a woman can have a copper-bearing IUD inserted any time within the first 12 days after the start of menstrual bleeding, at her convenience, and not just during menstruation (Question A). Meanwhile, a relatively larger proportion knew that copper-bearing IUDs should not always be removed if the user is diagnosed with pelvic inflammatory disease (70.7 %) (Question C), and that the most commonly used IUD, the CuT380a, was approved for 10 years of use after insertion (68.5 %) (Question E) [42] (Table 3).

**Table 3:** Questions assessing participating clinicians’ knowledge about contraception and IUDs

Question (True/False)	All *N* = 433	China *N* = 115	Kazakhstan *N* = 110	Laos *N* = 105	Mexico *N* = 103
a) A woman can have a copper-bearing intrauterine device (IUD) inserted any time within the first 12 days after the start of menstrual bleeding, at her convenience, not just during menstruation. *[True]*	34.4 % (149/433)	11.3 % (13/115)	53.6 % (59/110)	38.1 % 40/105)	35.9 % (37/103)
b) Spotting or light bleeding between menstrual periods is common during the first 3–6 months of copper-bearing intrauterine device (IUD) use. It is not harmful and usually decreases over time. *[True]*	74.5 % (322/432)	98.3 % (113/115)	66.7 % (72/108)	61.0 % (64/105)	70.2 % (73/104)
c) Copper-bearing IUD should always be removed if the intrauterine device (IUD) user is diagnosed with pelvic inflammatory disease (PID). *[False]*	70.7 % (304/430)	33.9 % (39/115)	87.2 % (95/109)	84.8 % (89/105)	80.20 % (81/101)
d) One follow-up visit after the first menses or 3–6 weeks following copper-bearing intrauterine device (IUD) insertion is sufficient. *[True]*	29.5 % 127/430)	8.7 % (10/115)	57.4 % (62/108)	35.6 % (37/104)	17.5 % (18/103)
e) The most commonly used IUD, the CuT380a, is approved for 10 years of use after insertion. *[True]*	68.5 % (296/432)	68.7 % (79/115)	71.3 % (77/108)	95.2 % (100/105)	38.5 % (40/104)
All answers correct	2.8 % (12/434)	0.9 % (1/115)	6.4 % (7/110)	3.8 % (4/105)	0 % (0/104)

Data show the percentage and fraction of respondents who correctly answered each question

Note that because of variations among sampling frames and a limited sample size, these results cannot, and should not, be compared across countries

Most participating clinicians reported that they “often” or “very often” undertook practices that were recommended and which matched the best available research evidence, including: performing a pelvic/genital examination before providing IUDs (80.4 %); recommending a follow-up visit after the first menses or 3–6 weeks following insertion when providing IUDs (83.2 %); recommending a follow-up visit when providing combined oral contraceptives (76.2 %); and screening for high blood pressure before providing combined oral contraceptives (67.0 %). However, a majority (66.6 %) of the respondents also reported that they “often” or “very often” performed a pelvic/genital examination before providing combined oral contraceptives, which is contrary to recommended practice (Table 4).

**Table 4:** Questions assessing participating clinicians’ practices relating to contraception and IUDs

Question (Frequency)	All *N* = 434	China *N* = 115	Kazakhstan *N* = 108	Laos *N* = 105	Mexico *N* = 106
a) Over the past 12 months, before providing intrauterine devices (IUDs), how often did you perform a pelvic/genital examination? [*Recommended practice*]	80.4 % (349/434)	88.7 % (102/115)	92.6 % (100/108)	67.6 % (71/105)	71.7 % (76/106)
b) Over the past 12 months, before providing combined oral contraceptives (COCs), how often did you perform a pelvic/genital examination? [*Contrary to recommended practice*]	4.64 % (20/431)	3.48 % (4/115)	0 % (0/106)	6.7 % (7/104)	8.49 % (9/106)
c) Over the past 12 months, when providing intrauterine devices (IUDs), how often did you recommend a follow-up visit after the first menses or 3–6 weeks following insertion? [*Recommended practice*]	83.2 % (356/428)	85.2 % (98/115)	89.3 % (92/103)	68.3 % (71/104)	89.6 % (95/106)
d) Over the past 12 months, when providing combined oral contraceptives (COCs), how often did you recommend a follow-up visit? [*Recommended practice*]	76.2 % (330/433)	70.4 % (81/115)	75.9 % (82/108)	66.3 % (69/104)	92.5 % (98/106)
e) Over the past 12 months, before providing combined oral contraceptives (COCs), how often did you screen for high blood pressure? [*Recommended practice*]	67.0 % (288/430)	44.7 % (51/114)	66.0 % (70/106)	67.3 % (70/104)	91.5 % (97/106)
All recommended practices	0.9 % (4/436)	0.9 % (1/115)	0.9 % (1/110)	0 % (0/105)	1.9 % (2/106)

Data show the percentage and fraction of respondents who over the previous 12 months engaged in the recommended practices described in the questions a,c,d,e either often or very often (vs. never, rarely, sometimes, and not applicable) and who never engaged in the non-recommended practice as described in the question b (vs. rarely, sometimes, often, very often, and not applicable)

Note that because of variations among sampling frames and a limited sample size, these results cannot, and should not, be compared across countries

The first ordinal logistic model identified two statistically significant factors associated with *knowledge* scores among clinicians related to family planning and IUD provision: 1) having a masters or doctorate degree (odds ratio [OR] 1.37, 95 % confidence interval [CI] 1.04–1.80); and 2) often reading scientific journals from high-income countries (OR 0.48, 95 % CI 0.30–0.77). The second ordinal logistic model identified five factors found to be associated with better self-reported *practices* related to family planning and IUD provision: 1) training in critically appraising systematic reviews (OR 1.69, 95 % CI 1.05–2.74); 2) training in the care of women seeking contraception (OR 1.72, 95 % CI 1.06–2.80); 3) having the perspective that research performed in their country is of above average or excellent quality (OR 1.72, 95 % CI 1.22–2.42); 4) being based in a facility or practice with an NGO as the operating authority (OR 1.65, 95 % CI 1.01–2.70); and 5) having the view that a higher quality of available research is important or very important to improving their work (OR 2.51, 95 % CI 1.05–6.01) (Table 5).

**Table 5:** Ordinal logistic models for factors associated with the log odds of demonstrating higher knowledge and better practices

Factor	Knowledge (*n* = 340)		Practices (*n* = 340)	
	OR	95 % CI	OR	95 % CI
Individual and practice characteristics				
Age^a^	0.96	(0.85, 1.08)	1.06	(0.81, 1.39)
Age squared^a^	1.00	(1.00, 1.00)	1.00	(1.00, 1.00)
Sex, female	0.99	(0.46, 2.09)	1.15	(0.47, 2.85)
Specialist physician	0.94	(0.66, 1.33)	1.50	(0.47, 4.81)
Time allocated to research ^b^	0.96	(0.92, 1.00)	0.98	(0.97, 1.00)
Master’s or doctorate degree	1.37	(1.04, 1.80)	0.90	(0.13, 6.50)
Training (since completed last degree) in:				
Acquiring systematic reviews through the Cochrane Library	0.88	(0.22, 3.44)	0.62	(0.22, 1.73)
Critically appraising systematic reviews	1.16	(0.54, 2.50)	1.69	(1.05, 2.74)
The care of women seeking contraception	0.87	(0.62, 1.23)	1.72	(1.06, 2.80)
Easy access to the internet	1.01	(0.73, 1.41)	0.98	(0.41, 2.32)
Able to read and write English well or very well	1.31	(0.70, 2.45)	1.16	(0.78, 1.71)
Working context				
Based in a facility or practice with an NGO as the operating authority	1.23	(0.27, 5.56)	1.65	(1.01, 2.70)
Located in an urban setting	1.15	(0.52, 2.55)	0.95	(0.68, 1.33)
Based in a hospital	0.91	(0.29, 2.85)	0.98	(0.27, 3.60)
Facility had anti-tuberculosis drugs available	0.46	(0.11, 1.85)	1.04	(0.85, 1.27)
Views and activities related to improving clinical practice				
Research performed in their own country is of above average or excellent quality	0.88	(0.69, 1.12)	1.72	(1.22, 2.42)
Trust somewhat or completely a systematic review of randomized controlled double-blind trials	1.21	(0.60, 2.46)	1.05	(0.71, 1.55)
Working with researchers or research groups to improve clinical practice or the quality of working life	0.89	(0.69, 1.15)	0.97	(0.65, 1.45)
Higher quality of available research is important or very important to improve their work	0.65	(0.27, 1.57)	2.51	(1.05, 6.01)
Used or read particular sources of evidence				
Clinical practice guidelines, protocols or decision-support tools	1.07	(0.66, 1.74)	1.14	(0.60, 2.17)
Cochrane Library	1.56	(0.53, 4.65)	0.84	(0.31, 2.29)
Scientific journals from high-income countries	0.48	(0.30, 0.77)	1.38	(0.72, 2.65)
Scientific journals from own country	0.84	(0.39, 1.79)	1.14	(0.75, 1.71)
Summaries of articles, reports, and reviews from public and not-for-profit health organizations	1.06	(0.72, 1.57)	1.21	(0.79, 1.87)
Thresholds				
k^1^	−2.09	(−4.63, 0.45)	3.18	(−2.37, 8.74)
k^2^	−0.53	(−3.19, 2.12)	4.53	(−1.27, 10.33)

*CI* confidence interval, *NGO* nongovernmental organization, *OR* odds ratio. Standard errors adjusted for four clusters (i.e., country). All regression models include country dummies (China is the reference country)

^a^Entered in regression models as continuous variables measured in years

^b^Entered in regression models as continuous variable measured in percent of time (0–100)

## Discussion

### Principal findings

The findings from this study suggest that significant gaps exist in clinicians’ knowledge and practices relating to family planning and IUD provision. From the population surveyed, only 12 of 434 (2.8 %) clinicians were able to correctly answer all five knowledge-testing questions. Similarly, only four of 436 (0.9 %) clinicians reported to “often” or “very often” perform the four recommended practices and never perform the one practice that was not recommended. These findings of less-than-optimal clinical knowledge and practices match those found in previous studies [19–27]. These gaps in IUD provision and family planning, however, may be a symptom of broader gaps in clinicians’ knowledge and practices for various health issues across low-, middle-, and high-income countries [55–71].

Through analyzing these knowledge and practice gaps, this study highlighted numerous factors associated with clinicians’ knowledge and/or practices related to family planning and IUD provision. Previous research findings have indicated that clinical training in IUD insertion can result in higher numbers of IUD insertion procedures [17] and fewer misconceptions about IUDs [21]. Accordingly, IUD training for clinicians will likely help to facilitate overall better practices in contraceptive and IUD provision in LMICs. Since gaps in knowledge and practices have varying degrees of consequences – with each gap resulting in more or less serious health and health system consequences – interventions targeting these gaps should be prioritized in whatever way is likely to produce the greatest health and social impact. Documented examples of knowledge and practice gaps and their health systems implications include the significant loss of life resulting from sub-optimal use of oral rehydration therapy for diarrhea and insecticide-treated bed nets for malaria prevention [3].

The ordinal logistic models presented in this study indicate that the knowledge and practices of clinicians may be disproportionately affected by certain factors more than others. Though it is possible that knowledge and practices are affected by different factors, the two regression analyses may also have been affected by social desirability biases. These biases would have likely affected the self-reporting of practices more so than in the answers to questions testing clinicians’ knowledge. Other explanations for the differing results could be the presence of confounders skewing the analysis, the models might have lacked necessary statistical power, and/or the insufficiency of the composite scores in representing clinicians’ actual “knowledge” and “practices”.

### Strengths and limitations of the study

There are five notable strengths of this study. The first is the diverse collection of data from four distinct LMICs which differ in characteristics such as life expectancy and contraception coverage rates. Second, the data collection from all four countries yielded very high response rates. Third, the data collected and examined is about the use of IUDs, a family planning method for contraception recommended by WHO and noted as essential for achieving global health goals [4, 5]. Fourth, the questionnaire was adapted from existing tools and assessed for reliability and validity [28]. Fifth, the knowledge and practice scores used in the analysis were calculated from a range of testing questions for which respondents were not given the correct answer. This metric is more objective than relying on the participating clinicians to self-evaluate whether they had “high” or “low” knowledge and/or practices – a method used in previous studies in this field.

Despite these strengths, the study also has four notable limitations. The first relates to the professional translation of study instruments into Mandarin, Russian, Lao and Spanish. It is possible that linguistic and cultural differences in the four countries could have affected participants’ understandings of particular translated survey questions. Second, the knowledge and practice scores were calculated from responses to only ten questions. Third, the questionnaire requested self-reported data to assess providers’ practices – a metric which is subject to social desirability bias. Self-reporting of practices may overestimate actual behaviour, and correspondingly result in an over-reporting of recommended practices [72]. Finally, the study faced resource constraints, causing a long delay in data analysis and manuscript writing and limiting the survey’s representative samples of the types of clinicians in each LMIC. For instance, the sample surveyed in Kazakhstan consisted of gynaecologists working in family planning, whereas respondents in Laos consisted of any clinician providing contraceptive care. As a result, the data obtained cannot be compared across the four countries. These variations also likely affected intra-country responses and, consequently, the data and results presented are not generalizable within country. The use of uniform study tools among uniform types of clinicians across each country would likely yield different results than those observed. To strengthen generalizability, studies resembling the current analysis should be conducted using representative samples of the same types of clinicians across countries.

### Policy implications

Clinicians serve as primary disseminators of health information and consequently hold considerable influence over patients’ health-related behaviours [1, 11–15]. In fact, recent literature surveying women’s attitudes toward contraception has revealed that physicians may be the source of greatest influence in the decision of which type of contraception is used [73]. With an acknowledgement of the significant impact that clinicians have on individuals and public health overall, efforts to improve clinicians’ knowledge and practices facilitate an opportunity to enhance family planning and maternal health globally. With the present study’s identification of gaps in clinicians’ knowledge and practices in this field, corresponding interventions to target such gaps are needed to help reduce the number of maternal deaths and contribute to the improvement of women’s reproductive health worldwide. With the implementation of programs focused on clinicians, it is possible that comparatively small investments could produce significant returns among larger populations. These programs should be informed by the large corpus of research on clinician training and behavioural change, including: educational meetings [74]; reminders [75]; audit and feedback [76]; outreach visits [77]; distribution of educational materials [78]; local opinion leaders [79]; and the freely available Health Systems Evidence database which contains these types of reviews [80]. Although, of course, even with the best training, clinicians would face many system-level barriers to optimal family planning services and IUD provision – including lack of availability, poor quality and high costs – which means that interventions targeting clinicians must be complemented by other interventions to strengthen the health systems in which they work. That is, policies and programs towards pressing health concerns, such as family planning and IUD provision, should be informed by evidence which considers the multifactorial interactions, as well as limitations, of the broader health system in which they are to be implemented [81].

### Future research

Future studies seeking to contribute to an understanding of clinicians’ knowledge and practices on family planning and IUD provision should seek to include representative samples of similar types of clinicians to enhance the generalizability of their findings across countries studied. In particular, funding to conduct research across larger, nationally representative samples of clinicians would allow for illustrative cross-sectional analyses. While this investigation has highlighted various factors that are associated with better knowledge and practices among clinicians, there remains the need for more evaluation and implementation of effective interventions to close existing know-do gaps.

## Conclusions

There persists a clear need for enhanced efforts and strategies targeted toward improving current knowledge and practices of clinicians regarding the use of contraceptives and IUDs. Key stakeholders and authorities, including policymakers, civil society leaders, donors and international organizations who are focused on improving the provision of family planning services, should evaluate existing strategies and consider novel interventions to improve the knowledge and practices of clinicians in this field. Multidimensional strategies across training institutes, clinics, policy forums, and stakeholder meetings are needed to cease the use of ineffective practices and instead facilitate the use of evidence-based practices. Targeted interventions should seek to improve the quality and accessibility of education and training programs for family planning clinicians, as well as assess and respond to domestic views on the usefulness of available research evidence. While this exploratory study highlights the gaps between knowledge and practice about family planning and IUDs among clinicians in four countries, further research is necessary to expand this investigation and continue the discussion on appropriate directions for family planning policy development and program implementation.
